# The Emerging Role of Innate Immunity in Chronic Kidney Diseases

**DOI:** 10.3390/ijms21114018

**Published:** 2020-06-04

**Authors:** Philip Chiu-Tsun Tang, Ying-Ying Zhang, Max Kam-Kwan Chan, Winson Wing-Yin Lam, Jeff Yat-Fai Chung, Wei Kang, Ka-Fai To, Hui-Yao Lan, Patrick Ming-Kuen Tang

**Affiliations:** 1 Department of Anatomical and Cellular Pathology, State Key Laboratory of Translational Oncology, Prince of Wales Hospital, The Chinese University of Hong Kong, Hong Kong 999077, China; philtang@link.cuhk.edu.hk (P.C.-T.T.); kamkwanchan@cuhk.edu.hk (M.K.-K.C.); jeffchung@link.cuhk.edu.hk (J.Y.-F.C.); wingyinlam@cuhk.edu.hk (W.W.-Y.L.); weikang@cuhk.edu.hk (W.K.); kfto@cuhk.edu.hk (K.-F.T.); 2 Department of Nephrology, Tongji Hospital, Tongji University School of Medicine, Shanghai 200065, China; idklaa@hotmail.com; 3 Li Ka Shing Institute of Health Sciences, and Department of Medicine & Therapeutics, The Chinese University of Hong Kong, Hong Kong 999077, China; hylan@cuhk.edu.hk

**Keywords:** chronic kidney disease, microenvironment, kidney fibrosis, macrophage–myofibroblast transition, inflammation

## Abstract

Renal fibrosis is a common fate of chronic kidney diseases. Emerging studies suggest that unsolved inflammation will progressively transit into tissue fibrosis that finally results in an irreversible end-stage renal disease (ESRD). Renal inflammation recruits and activates immunocytes, which largely promotes tissue scarring of the diseased kidney. Importantly, studies have suggested a crucial role of innate immunity in the pathologic basis of kidney diseases. This review provides an update of both clinical and experimental information, focused on how innate immune signaling contributes to renal fibrogenesis. A better understanding of the underlying mechanisms may uncover a novel therapeutic strategy for ESRD.

## 1. Introduction

Chronic kidney disease (CKD) is an emerging cause of morbidity and mortality worldwide. The global estimated prevalence of CKD is 13.4% (11.7%–15.1%) [1], affecting 26–30 million adults in the United States [2], 120 million adults in China, and causing renal replacement of 4.902 to 7.083 million patients. CKD defines as abnormalities of kidney structure or function caused by primary and secondary glomerular diseases, including glomerulonephritis, hypertension, and diabetic mellitus [3,4]. Notably, effective CKD treatment is still unavailable. 

Glomerulosclerosis and tubulointerstitial fibrosis are core manifestations of CKD, considered as the common fate of most chronic and progressive nephropathies toward end-stage renal disease (ESRD). In glomerulosclerosis, mesangial and endothelial cells play an important role in extracellular matrix (ECM) production by forming myofibroblasts [5]. In contrast, renal tubular epithelial cells and infiltrating immunocytes largely contribute to the ECM formation in tubulointerstitial fibrosis [6,7,8,9,10]. It is conceivable that glomerulosclerosis and tubulointerstitial fibrosis share similar disease mechanisms with minor differences. In general, collagen type IV deposits in the mesangial interstitial space and manifests as nodular changes in the glomeruli, whereas collagen type I deposits and manifests as diffuse changes in the tubulointerstitium [11]. To our knowledge, rare nephropathy initially presents glomerulosclerosis without interstitial fibrosis. Although the development of glomerular inflammation precedes interstitial fibrosis, tubulointerstitial fibrosis appears to be a uniform feature of progressive nephropathy, which best predicts renal failure [12]. 

Despite the number of causes, emerging evidence shows that renal inflammation plays a central role in the progression of kidney fibrosis [13,14,15,16]. The pro-fibrotic mechanisms of host immunity have been uncovered, contributing to the development of effective anti-fibrotic strategies. In this review, we summarized the updates of innate immunity in renal pathogenesis and their therapeutic implications in CKD (Table 1).

## 2. Inflammatory and Fibrotic Pathways in CKD

### 2.1. Inflammatory Pathway

#### 2.1.1. NF-κB Signaling

The NF-κB protein complex is the central regulator of the intricate inflammatory pathway network, responsible for the transcription of multiple inflammatory genes related to immunity, apoptosis, cell proliferation, and differentiation [32]. A systemic increase in inflammatory cytokines (IL-1β, TNF-α, LPS) activates NF-κB signaling associated with low-grade inflammation and chronic diseases, including CKD [33]. TNF-α and IL-1β interact with their respective receptors (TNFR1, IL-1R1) to activate NF-κB through phosphorylation of IKK (inhibitor of the κB kinase) with two catalytic (IKKα and IKKβ) and a regulatory subunit (IKKγ). In the canonical pathway, the activated IKK releases an NF-κB heterodimer (e.g., p50/RelA) from the inhibitory protein IκB for nuclear translocation. Different combinations of NF-κB dimers are complexed with various co-activators or transcription factors to regulate a variety of gene programs [34]. In the cohort study of type 2 diabetic nephropathy, NF-κB was specifically activated in renal tubular epithelial cells and significantly correlated with interstitial inflammation and proteinuria [35]. Furthermore, NF-κB also up-regulated in a number of renal diseases, including IgA nephropathy, immune-mediated inflammatory renal disease (crescentic glomerulonephritis, and lupus nephritis), minimal change disease, and membranous nephropathy [36]. NF-κB promotes inflammatory cytokines (e.g., IL-6, IL-1β) production to exacerbate renal inflammation associated with acute kidney injury (AKI) and diabetic nephropathy, which is largely suppressed by JSH-23 (NF-κB inhibitor) [37,38,39]. Interestingly, injured kidney-derived IL-6 can initiate the production of pro-inflammatory FGF23 in bone, which further stimulates liver cells to synthesize IL-6 and FGF23 for promoting CKD via fibroblasts activation [40,41,42]. Indeed, increasing FGF-23 levels are independently associated with an increased risk for incident kidney replacement therapy [43]. 

#### 2.1.2. Toll-like Receptors

Toll-like receptors (TLRs) are capable of detecting pathogens/damages for immune response. Pathogen-associated molecular patterns (PAMPs) in lipopolysaccharides (TLR4), peptidoglycans and lipoproteins (TLR1, 2, 6), viral RNA (TLR3), and unmethylated cytosine-guanosine dinucleotide (CpG)-DNA (TLR9) interact with specific TLRs to induce inflammatory cytokines and chemokines (TNF-α, IL-1β, IL-12, etc.) expression. The myeloid differentiation primary response protein 88 (MyD88) dependent pathway is the major TLRs signaling pathway [44,45]. TLRs also up-regulate co-stimulatory molecules in antigen-presenting dendritic cells (DC) [46]. In renal cells, TNF-α and IFN-γ up-regulated TLR2, TLR4, and TLR6 in mesangial cells, but down-regulated them in macrophages [47]. TLR2 promotes crescentic glomerulonephritis [48], whereas TLR4 promotes inflammation associated with ischemic AKI in diabetic mice, which can be rescued by the TLR4 specific inhibitor CRX-526 [49,50]. Specifically, TLR4 expressed in dendritic cells and macrophages contributes to autoimmune disorder systemic lupus erythematosus via T_H_1 polarization, which can be suppressed by conditional knockout of TLR4 and MyD88 on macrophages and DCs [51,52,53].

### 2.2. Fibrotic Pathway

#### 2.2.1. TGF-β1/Smads Signaling

Transforming growth factor-β1 (TGF-β1)/Smads signaling promotes renal fibrosis [54]. TGF-β1 is secreted as a latent complex. Proteases cleavage releases TGF-β1 to activate TGF-β receptor type I (TβRI) kinase for Smad2 and Smad3 phosphorylation and subsequent transcription [8,55,56]. Smad2/3 nuclear shuttling requires Smad4, which is essential for the Smad3 binding on the promoter region of collagen [57,58,59]. In addition, inhibitory Smad7 is degraded by the Smad ubiquitin regulatory factor (Smurf) to enhance TGF-β1/Smad3 signaling [15,60]. Among the Smads family, the pro-fibrotic role of Smad3 and anti-fibrotic role of Smad2 have been demonstrated by the loss-of-function studies in obstructive, diabetic, hypertensive, and acute toxic nephropathy in vivo [61]. Over the years, abundant evidence indicates the crucial role of Smads signaling dysregulation in fibrogenesis, extracellular matrix (ECM) synthesis, and myofibroblasts generation from macrophages, epithelial, and endothelial cells [55]. For example, global deletion and pharmacological inhibition of Smad3 protects against kidney fibrosis in UUO models [62,63]. Interestingly, conditional knockout of Tgfbr2 in proximal tubule (gGT-Cre) and tubular epithelial cells (Ksp-Cre) protect against AKI, whereas its loss in myeloid cells (LysM-Cre) promotes immunocytes (CD45^+^) infiltration associated with elevated pro-inflammatory cytokines, indicating the anti-inflammatory role of TGF-β1 [64,65,66]. Moreover, an activin A (ActA) antagonist, follistatin, blocks the TGF-β1-induced α-SMA and collagen I expression in renal fibroblasts and tubular cells, suggesting ActA as an autocrine factor for fibroblast activation in the development of kidney fibrosis [67,68]. To note, TGF-β1 also induces ActA to suppress the circulating level of the Wnt inhibitor Dickkopf one (DKK1) to further enhance fibrotic responses via cross-talk with Wnt signaling [69]. 

#### 2.2.2. Wnt/β-catenin Signaling

The Wnt family comprises a group of secretory lipid-modified signaling glycoproteins, and they interact with the Frizzled (Fzd) family and their co-receptors to activate cytoplasmic Disheveled (Dsh/Dvl) proteins for promoting canonical (β-catenin-dependent) or non-canonical (β-catenin-independent) signaling [70,71]. The persistent activation of Wnt/β-catenin signaling promotes chronic kidney disease and renal fibrosis. In an experimental CKD model, Wnt1 induces the prorenin receptor in kidney tubular epithelial cells to amplify the Wnt/β-catenin mediated pro-fibrotic genes expression (TGF-β, fibronectin, collagen I, plasminogen activator inhibitor-1, matrix metalloproteinase 7) [72,73]. Furthermore, Wnt/β-catenin signaling plays a crucial role in podocyte dysfunction, proteinuria, and glomerulosclerosis, particularly in diabetic nephropathy and focal segmental glomerulosclerosis [74,75]. Angiotensin II and high glucose activate Wnt/β-catenin signaling in podocyte, triggering the ubiquitin-mediated degradation of Wilms’ tumor 1 (WT1), a podocyte-specific transcription factor essential for their integrity and functions, resulting in albuminuria in mice [76,77,78]. Interestingly, condition knockout experiments showed that macrophages release Wnt7b to activate β-catenin signaling in tubular epithelial cells and dendritic cells for tissue regeneration and suppressing inflammation associated with Th1 differentiation, while the fibroblast-specific deletion of Wnt 4 suppresses renal fibrosis in UUO models [79,80,81], resulting in the anti-fibrotic effect of the CBP/β-catenin inhibitor PRI-724 [82,83]. These studies suggest the anti-inflammatory and pro-fibrotic role of Wnt/ β-catenin signaling in a cell-type specific manner.

#### 2.2.3. MAP Kinases Signaling

Mitogen-activated protein (MAP) kinases (ERK, p38, and JNK) can be activated by a wide range of stimulants such as growth factors, cytokines, and ligands for innate immune responses in a contextual manner. MAP kinases modulate TGF-β1 production and activation to promote renal fibrosis [8,84,85,86]. ERK and JNK activate activator protein-1 (AP-1) to promote TGF-β1-induced pro-fibrotic factors production, which contributes to the anti-fibrotic effect of the JNK inhibitor (CC-401) [87,88,89]. In mesangial cells, angiotensin II induces p38- and JNK-dependent thrombospondin-1 (TSP-1) expression for latent TGF-β1 activation [90]. Moreover, MAP kinases can modulate Smad signaling via the canonical pathway [8]. TGF-β1 also can induce ERK- and p38-dependent Smad3 activation, facilitating ECM synthesis, fibroblasts recruitment, and differentiation [91]. In addition, JNK promotes renal inflammation via the AP-1-mediated transcription of leukocyte adhesion molecules in endothelial cells and pro-inflammatory cytokines production in epithelial cells to facilitate leukocytes infiltration and recruitment, respectively [92]. 

#### 2.2.4. JAK/STAT Signaling

The Janus kinase (JAK) family (JAK1, JAK2, JAK3, and TYK2) and signal transducer and activator of transcription (STAT) family (STAT1–STAT4, STAT5A, STAT5B, and STAT6) [93,94] transduce signals for numerous growth factors and cytokines, including families of interferon (IFN), gp130, and γC in an isoform-specific manner [95]. Upon ligand binding, JAK phosphorylates the cytokine receptor via the tyrosine residues of the cytoplasmic domain, which in turn recruits and activates STAT for nuclear translocation to bind on their target genes. Among JAKs/STATs, the pro-fibrotic role of JAK2/STAT3 is observed in experimental models and clinical studies of renal dysfunction and fibrosis [96]. Berthier et al. found that JAK/STAT signaling up-regulated in mice models and patients with type 2 diabetic nephropathy [97]. Yokota et al. revealed that STAT3 activation (p-STAT3 (Tyr705)) mediates pro-inflammatory and fibrotic genes expression in Alport syndrome [98]. Bienaimé et al. further demonstrated that STAT3 in tubular cells promotes interstitial fibrosis and α-smooth muscle actin (α-SMA) expression in 3/4 nephrectomy models [96]. On the contrary, Lan et al. showed the renoprotective role of STAT3 in acute aristolochic acid nephropathy [99], suggesting the contextual role of JAK/STAT signaling. Furthermore, conditional knockout models reveal the anti-inflammatory action of Stat3 in myeloid cells but pro-inflammatory role in T cells and epithelial cells [100,101,102]. Notably, the JAK1/2 inhibitor baricitinib effectively suppresses the progression of diabetic kidney disease, implicating the therapeutic implication of JAK/STAT signaling in renal fibrosis [103]. 

## 3. Roles of Innate Immunity in CKD

**Figure 1:**
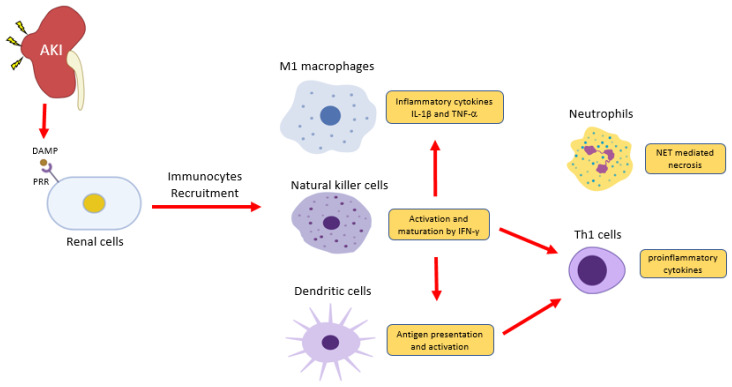
Inflammatory cells in acute kidney injury (AKI). Renal cells damage releases damage-associated molecular patterns (DAMPs) to induce inflammation via pattern recognition receptors (PRRs). Natural killer cells release IFN-γ to induce classical activation of macrophage (M1)- producing pro-inflammatory cytokines IL-1 and TNF2 and induce dendritic cell maturation for pro-inflammatory Th1 differentiation. DAMPs also trigger the release of a neutrophils extracellular trap (NET) to further recruit inflammatory cells and induce renal cell death.

Renal injury eventually progresses to CKD under unresolved inflammation [104]. Upon kidney injury, damage-associated molecular patterns (DAMPs) trigger inflammatory responses, resulting in immunocytes infiltration predominantly to neutrophils, macrophages, and natural killer cells [105] (Figure 1). Together with resident dendritic cells, innate immune cells thus take corresponding roles in damage and repair on the site of injury. Recent studies reveal that renal cell death releases endogenous cytokines, chemokines, oxidative stress, and DAMPs, which largely promotes infiltration and activation of immune cells that result in CKD [106,107,108,109].

### 3.1. Neutrophils

Inflammation triggers the production of reactive oxygen species (ROS) and serine proteases of neutrophils upon adhesion to the injured site, which are believed to combat bacterial infection [106] and trigger the formation of neutrophil extracellular traps (NETs) [107]. NETs are found in acute tubular necrosis as a unique form of cell death, where intracellular membranes are degraded due to the histones and granule proteins attached on the ejecting chromatin [108]. Chromatin-released NETs also act as DAMPs to elicit inflammatory and cytotoxic effects, and Singh et al. reported that the histones released from NETs could enter and kill renal cells by nonspecific DNA and RNA binding. Besides, extracellular histones can ligate to the toll-like receptors −2 and −4 and nucleotide-binding domain (NOD)-like receptor protein 3 for inducing inflammasome [109]. Altogether, NETs generate an auto-amplification loop of inflammation to accelerate tubular necrosis, therefore causing irreversible damage to the nephrons [18].

### 3.2. Dendritic Cells

DCs are antigen-presenting cells. They are derived from bone marrow as an immature state as precursor DCs, then circulate into peripheral blood for foreign and pathogenic antigens detection [110]. Upon inflammatory stimuli at the injured site, endogenous DAMPs and PAMPs trigger the maturation of DCs for antigen presentation, cytokines, and co-stimulatory molecules expression. Interestingly, inflammatory stimuli transform dendritic cells to be a distinct subset called inflammatory DCs (infDCs), which activate T cells for promoting inflammation [111,112]. The infDCs secrete IL-1, TNF-α, IL-12, and IL-23 to stimulate IL-17 production in CD4^+^ and CD8^+^ T cells in vivo [112]. Inhibition of Flt3, a ligand for cross-presentation between DCs and T cells, significantly reduces infiltration and proliferation of CD4^+^ and CD8^+^ T cells, therefore alleviating kidney inflammation in experimental adriamycin nephropathy models [22]. These findings suggest DCs could activate adaptive immunity and the autoimmune response for facilitating renal inflammation.

### 3.3. Natural Killer Cells

Natural killer (NK) cells are modulators of innate and adaptive immune responses. Their surface activating and inhibitory receptors are responsible for regulating NK cells’ activities upon interactions to target cells, complementary and antagonist pathways that are initiated to trigger NK cells to secrete cytokines and chemokines to regulate neighboring immune cells [113,114]. Studies found that NK cells could promote Th1 polarization of CD4^+^ T cells and maturation of DCs through IFN-γ [115]. In addition, the activated NK cells are capable of eliminating DCs that fail to complete their maturation [116]. It is believed that NK cells modulate differential immune responses depending on the cytokine environment. Recent in vitro studies found that exposure of NK cells to exogenous IL-12 would induce strong cytolytic activity against immature DCs; in contrast, IL-4-conditioned NK cells would generate DCs favoring T cell polarization or Th2 priming [117]. Thus, NK cells regulate DCs and T cells in the renal microenvironment.

### 3.4. Macrophages

Macrophages are highly plastic, and they contribute to every stage of CKD, from renal inflammation to fibrosis. In experimental CKD models, endogenous DAMPs and PAMPs induce M1 pro-inflammatory macrophages [118,119,120,121], therefore producing inflammatory cytokines IL-1β and TNFα to promote renal inflammation [25,26,122]. Nevertheless, studies also reported that macrophage infiltration correlates with active fibrotic lesions, supported by the significant reduction in renal fibrosis in IRI and UUO models under macrophage depletion [123,124]. M1 macrophages induce chronic renal inflammation, resulting in collagen and extracellular matrix deposition [125]. During CKD progression, M1 is gradually replaced by the reparative M2 phenotype [126]. The M1/M2 transition is evident and characterized by a time-dependent exchange of M1/M2 markers and the existence of their intermediate population, detected by single-cell sequencing analysis in AKI, glomerular disease, and UUO models [127,128,129]. Clinical studies of diabetic nephropathy [130] and kidney transplantation [131] showed that M2 macrophages localize at the fibrotic areas and actively produce pro-fibrotic molecules IL-1, PDGF, MMP-2/9/12, and galectin 3 [27,28]. Moreover, bone marrow-derived macrophages (BMDM) could further differentiate into α-SMA^+^ myofibroblasts locally in injured kidney under unresolved inflammation for promoting renal fibrosis [31,131]. These studies demonstrate the pro-fibrotic role of M2 macrophages in renal fibrosis. 

## 4. Novel Pathogenic Mechanism: Macrophage-Myofibroblast Transition

**Figure 2:**
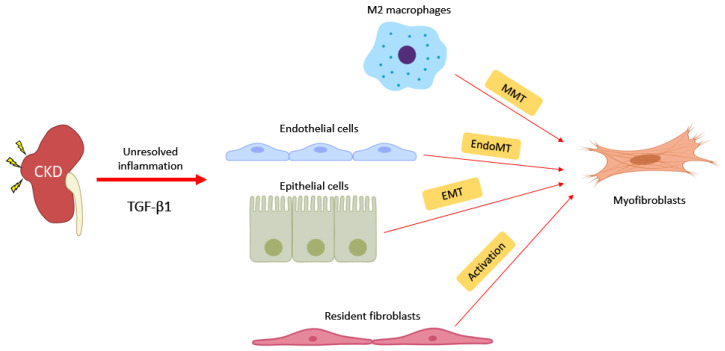
Macrophages promote chronic kidney disease (CKD) progression from AKI to fibrosis. During unresolved inflammation, TGF-β1 further differentiates M2 macrophages into myofibroblasts. Therefore, TGF-β1 triggers myofibroblasts formation via the macrophage–myofibroblast transition (MMT), endothelial–mesenchymal transition (EndoMT), epithelial–mesenchymal transition (EMT), and fibroblast activation in the fibrotic kidney.

Myofibroblasts are activated fibroblasts featured with an α-SMA expression and pathogenic collagen production during tissue fibrosis [132]. Previous studies identified that BMDM could further differentiate into α-SMA^+^ myofibroblasts via a novel mechanism, namely macrophage–myofibroblast transition (MMT) [30,31] (Figure 2). MMT cells co-expressing macrophage (CD68^+^) and myofibroblast (α-SMA^+^) markers were detected and positively correlated with the abundance of myofibroblasts in active chronic renal allograft injury [30]. The role of MMT in tissue scarring was demonstrated in vivo, and macrophage-lineage myofibroblasts and their intermediate cells (F4/80^+^ α-SMA^+^) exist in fibrotic kidney of Crim1 hypomorphic mice, UUO, and chronic renal allograft rejection mouse models [133]. These studies provide experimental evidence for the pathogenic role of MMT in renal fibrosis. 

MMT is driven by the TGFβ1/Smad3 signaling pathways in fibrotic kidney [8,55]. A deficiency of Smad3 protects against myofibroblast formation and renal fibrosis in various experimental mouse kidney injury models [134,135]. Src is the upstream of Smad3, regulating fibroblast proliferation and renal fibrosis [136]. Unexpectedly, we recently revealed that Src can also serve as the direct target gene of TGFβ1/Smad3 signaling for promoting MMT via a regulatory gene network in UUO-injured kidney [137]. Thus, MMT may represent a novel therapeutic target for blocking CKD development.

## 5. The Perspective of Immunosuppressive for CKD

### 5.1. Clinical Ready Immunotherapy for CKD

Indeed, a number of immunosuppressive agents are already prescribed for immune-mediated kidney diseases including AKI, CKD, and GvHD. The use of immunosuppressors in CKD is still under debate, only patients with massive proteinuria will receive these treatments to balance the benefit and risk of immunosuppression. In this section, we will summarize the potential therapeutic strategies according to the immune cell targets.

#### 5.1.1. T cell-targeted Therapy

T cell targeted therapy works in three ways by targeting 1) the interaction between the T cell receptor complex and antigen-presenting cells (APC); 2) co-stimulatory signals on T cell/APC; and 3) cytokine driven activation and proliferation [138]. For example, calcineurin inhibitors (CNIs) are commonly used in nephropathy treatment, which prevents the nuclear translocation of nuclear factor of activated T cells (NFAT). However, dose-dependent renal toxicity, dysfunction, and failure were observed in nephropathy and post-renal transplantation after prolonged treatment with the CNIs cyclosporin and tacrolimus and an investigational agent voclosporin [139,140]. New extended-release formulations and CNIs may overcome these side effects [141,142]. T cells express CTLA4 (cytotoxic T lymphocyte-associated protein 4) to competitively inhibit the co-stimulatory signals CD80/CD86 on APC [143]. Recombinant CTLA4-Ig has been developed and approved for rheumatoid arthritis and focal segmental glomerulosclerosis (FSGS) with CD80^+^ podocytes. 

#### 5.1.2. B cell-targeted Therapy

B cell-targeted therapy suppresses the maturation and differentiation of B cells into antibody-producing plasma cells by inhibiting CD20, CD22, and B cell activating factor (BAFF) to alter the course of autoimmune and alloimmune diseases’ progression [138]. Rituximab targets CD20 to prevent B cell proliferation and induce apoptosis via both complement-dependent and -independent mechanisms. Recently, rituximab is widely used in idiopathic membranous nephropathy (IMN), FSGS, and lupus nephritis with anecdotal success [144]. Belimumab improves the renal condition of systemic lupus erythematosus by suppressing B cell survival and differentiation via targeting the soluble BAFF [145]. Bortezomib inhibits the antibody production of mature plasma cells, which is effective in renal function improvement [146]. However, direct evidence of T cell- or B cell-targeted agents in renal fibrosis is still lacking.

#### 5.1.3. Mesenchymal Stem Cells (MSCs) Therapy

Mesenchymal stem cells (MSCs) therapy is a new therapeutic strategy for end-stage renal disease (ESRD) [147]. MSCs are pluripotent stem cells capable of differentiating into various tissue types for diverse biological functions including immune regulation. Several studies explored the feasibility of MSCs for renal fibrosis therapy. MSCs transfusion effectively suppressed renal fibrosis in experimental models by inhibiting both the pro-inflammatory and pro-fibrotic signaling pathways, including TGF-β1/Smad3, TLR4/NK-κB, and ERK [147,148,149]. MSCs-based therapy facilitates tissue repair in experimental models and patients with AKI [150], and diminishes kidney fibrosis in obstructive nephropathy when combined with a low dose of tacrolimus [151]. More importantly, MSCs-based therapy significantly decreases the rate of solute transport across the peritoneal membrane in peritoneal dialysis patients, resulting in better clinical outcomes [152]. MSCs therapies have demonstrated their renal protective effects in mice models and clinical trials, and there are on-going clinical trials which should further validate the effectiveness of MSCs therapy in ESRD. 

#### 5.1.4. Chimeric Antigen Receptor T (CAR T) Cells Therapy

The chimeric antigen receptor (CAR) T cell is an engineered cytotoxic T cell activated by the direct recognition of an antigen without a major histocompatibility complex (MHC). T cells are obtained from patients by leukapheresis, then expanded and transduced with viral vectors encoding the fusion protein CAR for recognizing the targeted antigen. Finally, these CAR T cells are infused back to the patients [153,154]. CAR T cell therapy is usually accompanied by immune effector cell-associated neurological syndrome (ICANS) and cytokine release syndrome (CRS), while direct renal toxicity is not acknowledged. In a retrospective review, Gupta et al. found that CAR T therapy restored the kidney function of 46 patients with non-Hodgkin lymphoma to the baseline within 30 days and helped to recover from AKI [155]. Kitching et al. suggested that CAR T cells could be applied to autoimmune diseases, including kidney transplantation, by loading CAR into cytotoxic T cells to eliminate autoreactive B cells and into regulatory T cells to suppress the autoimmune response locally [156].

#### 5.1.5. Inflammatory Reflex Targeted Therapy

In AKI, the central nervous system (CNS) has been reported to regulate the immune response via inflammatory reflex. Upon injury, danger stimuli including DAMPs activate pattern recognition receptors (PRRs) on the local afferent vagus nerve to generate a signal transmitted through the CNS and efferent vagus nerve to the splenic nerve that stimulates the release of noradrenaline in the spleen [157,158]. Noradrenaline activates choline acetyltransferase positive T cells (ChAT^+^) via the β2-adrenergic receptor, leading to the subsequent release of acetylcholine, which binds to the α7 nicotinic acetylcholine receptor (α7nAChR) on macrophages to suppress pro-inflammatory cytokines production [159]. Therapeutic strategies mimicking the efferent arm of the inflammatory reflex by applying pulsed ultrasound to the spleen or α7nAChR agonists successfully protected kidney against AKI in experimental models, suggesting neuro-immune control is a potential target to suppress renal inflammation [160,161]. 

### 5.2. Effect of Immunotherapy in Experimental CKD Models

Leukocyte infiltration plays a pivotal role in the pathogenesis of renal fibrosis. Upon injury, infiltrated leukocytes secret pro-inflammatory cytokines to amplify the inflammatory responses, the unresolved inflammation eventually resulting in renal fibrosis as well as the loss of kidney functions. Therefore, therapies preventing leukocytes infiltration have been widely studied. A study reported that circulating T cell clones may directly activate renal epithelial cells or promote a T/B cell population with autoimmune reactive properties against kidney cells [162]. Activating the AT1 receptor on T cells effectively suppresses renal fibrogenesis by inhibiting Th1 differentiation and renal accumulation of pro-fibrotic macrophages [163]. Furthermore, macrophage infiltration and collagen deposition were attenuated by genetic and anti-CD20-mediated B cell depletion in mice with obstructive nephropathy [164]. Macrophages are versatile, which participated in the pathogenesis of renal diseases as well as vital physiological functions (e.g., tissue repair, immune regulation, and defense against pathogen). To minimize the side effects, specific strategies were developed to suppress pathogenic macrophage infiltration, polarization, and myofibroblast transition via chemokines (CCL2, CCL5, CXCL16, and CCL21) and their receptors, Src, JAK-STAT, and TGFβ1/Smad3 signaling inhibition, respectively, to attenuate the progression of renal fibrosis. These macrophage-targeting strategies may be translated by further clinical investigation [9].

## 6. Conclusions

Patients who recover from acute kidney injury are likely to progress into chronic kidney disease, and eventually lead to end-stage renal diseases. Innate immunity is the first line of inflammatory cells infiltrated into the injured kidney, performing diverse functions, from amplifying the inflammatory response to renal repair. However, they also largely contribute to the development of renal fibrosis during the transition of AKI into CKD under unresolved renal inflammation. Therefore, a better understanding of the underlying mechanism may uncover effective therapeutic strategies for blocking the progression of CKD. 

## Figures and Tables

**Table 1:** Summary of the role of innate immune cells in the pathogenesis of kidney diseases.

Diseases	Models	Role of Inflammatory Cells	Ref.
**Neutrophils**			
AKI	Renal I/R injury	Neutrophils release extracellular DNA (NET) to stimulate inflammation via toll-like receptor signaling and platelet activation.	[17]
AKI	Renal I/R injury	Neutrophils induce tubular necrosis via PAD-mediated NET formation	[18]
Glomerulo-nephritis	Anti-GBM Nephritis	Histones released by neutrophils induce glomerular vascular injury by direct killing of endothelial cells	[19,20]
**Dendritic cells**			
Focal segmental glomerulo-sclerosis	Adriamycin nephropathy	CD103^+^ dendritic cells activate CD8^+^ T cells to induce apoptosis of tubular epithelial cells and inflammatory cytokines (TNF-α and IFN-γ) release.	[21,22]
**Natural killer cells**			
Lupus nephritis	MRL/MpJ, MRL/lpr mice	Infiltrated NK cells secret IFN-γ to promote renal inflammation	[23]
AKI	Renal I/R injury	Activated NK cells induce kidney injury via attacking tubular epithelial cells	[24]
**Macrophages**			
Crescentic glomerulonephritis	Anti-GBM Nephritis	Macrophages express pro-inflammatory molecules (tumor necrosis factor, MMP-12, CCL2, and IL-12) in crescentic injury.	[25,26]
Renal fibrosis	Unilateral Ureter Obstruction	Alternative activated macrophage produces pro-fibrotic molecules (MMPs and Galectin 3) for the development of renal fibrosis	[27,28]
Renal fibrosis	Unilateral Ureter Obstruction Kidney Trans-plantation	Alternative activated macrophage further transits into α-SMA^+^ collagen-producing myofibroblast for extensive extracellular matrix deposition	[29,30,31]

## Abbreviations

ActA	Activin A
α7nAChR	α7 Nicotinic Acetylcholine Receptor
AKI	Acute Kidney Injury
AP-1	Activator Protein-1
APC	Antigen-Presenting Cell
α-SMA	Alpha-Smooth Muscle Actin
BAFF	B-Cell Activating Factor
CAR-T	Chimeric Antigen Receptor T Cells
CD-	Cluster of Differentiation-
ChAT+	Choline Acetyltransferase Positive T Cells
CKD	Chronic Kidney Disease
CNIs	Calcineurin Inhibitors
CTLA4	Cytotoxic T-Lymphocyte-Associated Protein 4
DAMPs	Damage-Associated Molecular Patterns
DC	Dendritic Cell
DKK1	Dickkopf One
EMT	Epithelial-Mesenchymal Transition
EndoMT	Endothelial-Mesenchymal Transition
ERK	Extracellular-Signal-Regulated Kinase
ESRD	End-Stage Renal Disease
FGF-23	Fibroblast Growth Factor 23
FSGS	Focal Segmental Glomerulosclerosis
gGT1-Cre	Gamma-Glutamyltransferase 1, Promoter. Cre
GVHD	Graft Versus Host Disease
IFN-	Interferon-
IKK	Iκb Kinase
IL-	Interleukin-
IMN	Idiopathic Membranous Nephropathy
IRI	Ischemia/Reperfusion Injury
JAK	Janus Kinase
JNK	C-Jun N-Terminal Kinase
Ksp-Cre	Cadherin 16 Promoter. Cre
LAP	Latency-Associated Peptide
LPS	Lipopolysaccharides
LTBP	Latent TGF-β Binding Proteins
MAPK	Mitogen-Activated Protein Kinases
MCP-1	Monocyte Chemoattractant Protein-1
MHC	Major Histocompatibility Complex
MMP-	Matrix Metallopeptidase-
MMT	Macrophage-To-Myofibroblast Transition
MSCs	Mesenchymal Stem Cells
MYD88	Myeloid Differentiation Factor 88
NETs	Neutrophil Extracellular Traps
NFAT	Nuclear Factor of Activated T-Cells
NF-kB	Nuclear Factor Kappa-Light-Chain-Enhancer of Activated B Cells
NK	Natural Killer Cell
PAMPs	Pathogen-Associated Molecular Patterns
PDGF	Platelet-Derived Growth Factor
ROS	Reactive Oxygen Species
SMADs	Mothers Against Decapentaplegic Homologs
SRC	Proto-Oncogene Tyrosine-Protein Kinase Src
STAT	Signal Transducer and Activator Of Transcription
TCR	T-Cell Receptor
TGF-β	Transforming Growth Factor-Βeta
TGFBR2	Transforming Growth Factor Beta Receptor 2
Th1/2	T Helper Type 1/2
TLRs	Toll-Like Receptors
TNF-	Tumor Necrosis Factor
TSP-1	Thrombospondin-1
TΒRI	TGF-β Receptor Type I
UUO	Unilateral Ureter Obstruction
WNT	Wingless-Type MMTV Integration Site Family
